# Early Effects of Porcine Placental Extracts and Stem Cell-Derived Exosomes on Aging Stress in Skin Cells

**DOI:** 10.3390/jfb15100306

**Published:** 2024-10-15

**Authors:** Takaaki Matsuoka, Katsuaki Dan, Keita Takanashi, Akihiro Ogino

**Affiliations:** 1 Omotesando Helene Clinic, Minato-ku 107-0062, Tokyo, Japan; 2 Department of Pathophysiology, Yokohama University of Pharmacy, Yokohama-shi 245-0066, Kanagawa, Japan; kdan@iyakushigen.jp (K.D.); keita.takanashi@yok.hamayaku.ac.jp (K.T.); 3 Division of Research and Development, Research Organization of Biological Activity, Shibuya-ku 150-0001, Tokyo, Japan; 4 Department of Plastic and Reconstructive Surgery, Toho University Omori Medical Center, Ota-ku 143-8541, Tokyo, Japan; akihiro.ogino@med.toho-u.ac.jp

**Keywords:** placental extracts, aging, human mesenchymal stem-cell-derived exosomes, senescence

## Abstract

The initial efficacy of placental extracts (Pla-Exts) and human mesenchymal stem-cell-derived exosomes (hMSC-Exo) against aging-induced stress in human dermal fibroblasts (HDFs) was examined. The effect of Pla-Ext alone, hMSC-Exo alone, the combined effect of Pla-Ext and hMSC-Exo, and the effect of hMSC-Exo (Pla/MSC-Exo) recovered from cultures with Pla-Ext added to hMSC were verified using collagen, elastin, and hyaluronic acid synthase mRNA levels for each effect. Cells were subjected to photoaging (UV radiation), glycation (glycation end-product stimulation), and oxidation (H_2_O_2_ stimulation) as HDF stressors. Pla-Ext did not significantly affect normal skin fibroblasts with respect to intracellular parameters; however, a pro-proliferative effect was observed. Pla-Ext induced resistance to several stresses in skin fibroblasts (UV irradiation, glycation stimulation, H_2_O_2_ stimulation) and inhibited reactive oxygen species accumulation following H_2_O_2_ stimulation. Although the effects of hMSC-Exo alone or the combination of hMSC-Exo and Pla-Ext are unknown, pretreated hMSC-Exo stimulated with Pla-Ext showed changes that conferred resistance to aging stress. This suggests that Pla-Ext supplementation may cause some changes in the surface molecules or hMSC-Exo content (e.g., microRNA). In skin cells, the direct action of Pla-Ext and exosomes secreted from cultured hMSCs pretreated with Pla-Ext (Pla/MSC-Exo) also conferred resistance to early aging stress.

## 1. Introduction

Despite daily exposure to external environmental stresses, skin cells (keratinocytes, fibroblasts, dermal stem cells, etc.) cooperate to maintain the extracellular matrix environment, ensuring an ongoing stress response. The major contributors to skin aging include photoaging (UV radiation), glycation (advanced glycation end-product [AGE] stimulation), and oxidation (H_2_O_2_ stimulation). Resistance to these stresses is one way of combating skin aging, with the inhibition of intracellular reactive oxygen species (ROS) accumulation being crucial [1,2]. Recently, in vivo cultured stem cells from regenerative medicine research have been incorporated in skin anti-aging research, and the skin anti-aging effects of extracellular vesicles, such as mesenchymal stem-cell-derived exosomes (hMSC-Exo), have been investigated [3,4]. Stem cells have been shown to have a function called the homing effect. According to this theory, when they receive rescue signals from damaged tissues or cells, they rush to the affected area and regenerate into those cells. It has been shown that the signal transduction is carried out by stem-cell-derived exosomes, which also contribute to wound healing in the skin [5,6]. However, since it takes 3 to 4 months for stem cells to regenerate, it is important to enhance the role of stem-cell-derived exosomes that can act earlier in order to adapt them to cellular aging. While an anti-aging effect that can improve aged cells is a desirable effect, it is thought that resistance to early aging stress may also have a preventive effect.

Human placental extracts (hPLa) have been widely used in traditional medicine, including Chinese medicine, for wound healing [7,8]. hPLa is considered a “natural reservoir” because it contains numerous bioactive materials, including vitamins, amino acids, peptides, growth factors, and trace elements [9]. Subcutaneous and intramuscular hPLa injections have been used clinically to treat hepatic diseases and menopausal disorders [10]. Moreover, hPLa is used clinically as an anti-wrinkle agent, mainly in Asian countries, due to its antioxidant, cellular growth, and collagen synthesis effects [11,12]. However, since the use of hPLa as a medicinal product is limited in some countries, such as Japan, porcine placental extracts (Pla-Exts) have been recently developed as a substitute for hPLa and are used as a raw material in healthy foods and cosmetics [13]. Pla-Ext reportedly has antioxidant, immunomodulatory, and immunopotentiating effects [14,15,16]. Evidence suggests that Pla-Ext stimulation increases cell numbers and collagen and elastin production in human dermal keratinocytes, and Pla-Exts can penetrate epidermal tissue to reach fibroblasts and skin stem cells in a three-dimensional skin model (the data are being prepared for publication). hMSC-Exos have also been demonstrated to repair fibroblast damage under H_2_O_2_ water stress [17].

Here, we evaluated the response elicited by the Pla-Ext treatment of cultured human dermal fibroblasts (HDFs) to verify its efficacy against human skin aging and whether it confers resistance to damage when HDFs are subjected to several types of aging stress. To determine the effects of the control (purified water [DW]), hMSC-Exo alone, Pla-Ext and hMSC-Exo combined, and hMSC-Exo (Pla/MSC-Exo) recovered from culture broth containing Pla-Ext added to hMSCs (preconditioning), HDFs were subjected to photoaging (UV radiation), saccharification (AGE stimulation), and oxidation (H_2_O_2_ stimulus).

Dermal fibroblasts have been shown to be capable of synthesizing collagen, elastin, and dermal hyaluronan, as indicators for several stress responses. Therefore, the mRNA levels of these proteins were measured in the present study [18,19]. This study aimed to determine the initial mechanism of action underlying the anti-aging effect of Pla-Ext on human skin. In addition, we also investigated whether the mechanism of action was altered by MSC-Exo obtained from preconditioning, where Pla-Ext acts directly on hMSC-Exo.

## 2. Results

### 2.1. Effects on Normal HDFs

**Figure 1:**
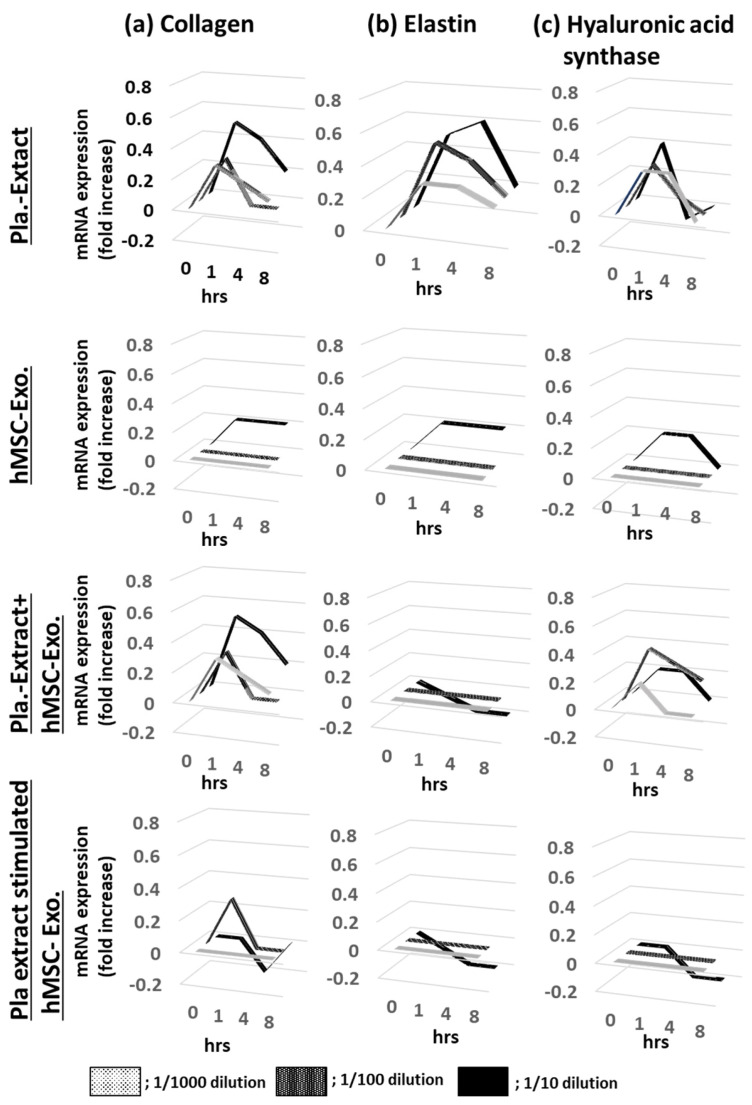
Effects of Pla-Ext on (**a**) collagen, (**b**) elastin, and (**c**) hyaluronic acid synthase mRNA expression levels in normal human dermal fibroblasts. Pla-Ext, placental extract; hMSC-Exo, human mesenchymal stem-cell-derived exosomes; Pla extract-stimulated hMSC-Exo; placenta extract-stimulated human mesenchymal stem-cell-derived exosomes.

**Figure 2:**
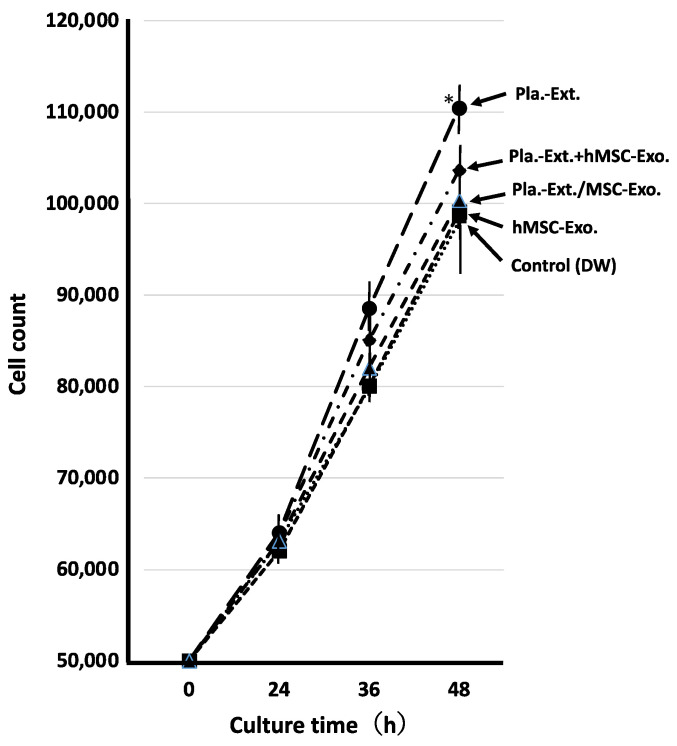
Effects of Pla-Ext on normal human dermal fibroblast proliferation. Pla-Ext, placental extract; hMSC-Exo, human mesenchymal stem-cell-derived exosomes; Pla extract-stimulated hMSC-Exo; placenta extract-stimulated human mesenchymal stem-cell-derived exosomes. * *p* < 0.05 vs. control (DW).

HDFs were treated with five additives, and the mRNA expression levels for collagen, elastin, and hyaluronic acid synthase were assessed over time (Figure 1a–c). After adding Pla-Ext, the mRNA expression levels tended to increase in a concentration-dependent manner from 1 to 4 h, but none significantly increased. No mRNA variations were observed in the group treated with hMSC-Exo alone. In addition, the mRNA levels following Pla-Ext and hMSC-Exo combination stimulation or hMSC–supplement exosome treatment of normal skin cells did not vary significantly. Furthermore, the effects of the five treatments on cell proliferation were examined. All treatment groups exhibited higher proliferation compared to the controls, with the Pla-Ext-treated group demonstrating the highest growth potential (a 13% increase compared to the controls at 48 h; *p* < 0.05) (Figure 2).

### 2.2. Damage Resistance in HDFs Subjected to Photoaging Stimuli (UV Radiation)

**Figure 3:**
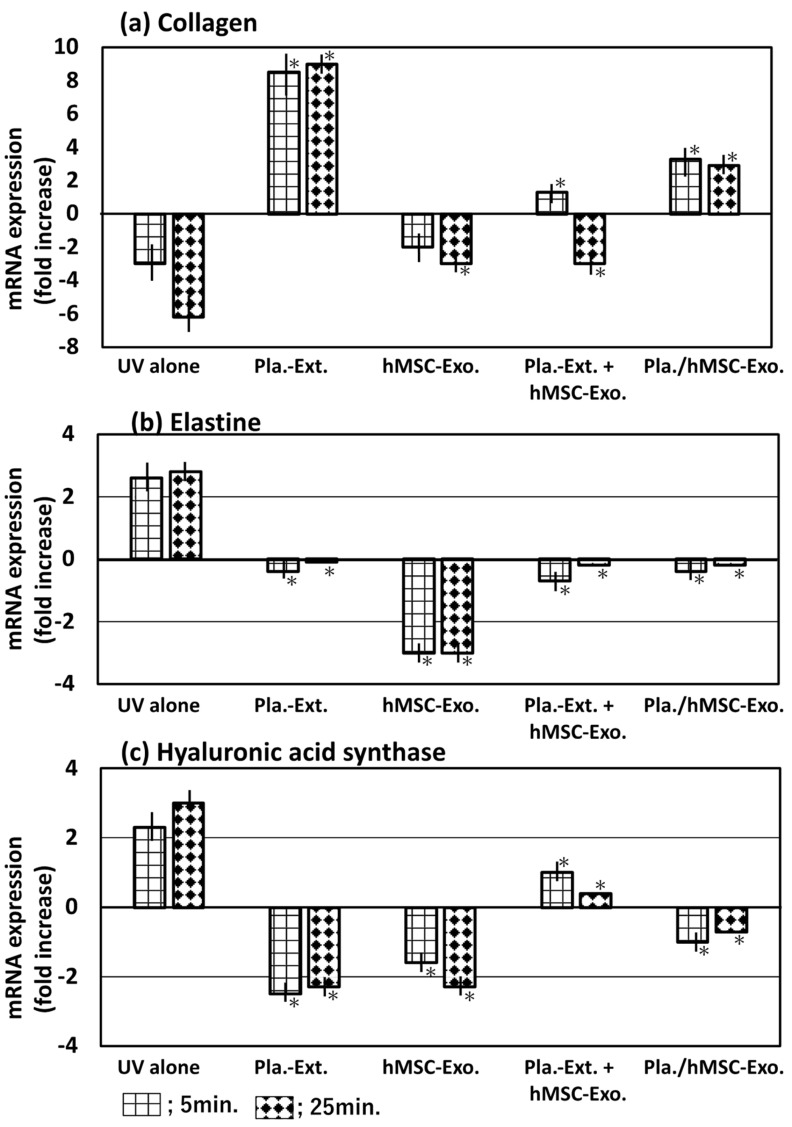
Effects of Pla-Ext on (**a**) collagen, (**b**) elastin, and (**c**) hyaluronic acid synthase mRNA expression levels in UV-treated human dermal fibroblasts (5 or 25 min). Pla-Ext, placental extract; hMSC-Exo, human mesenchymal stem-cell-derived exosomes; Pla extract-stimulated hMSC-Exo; placenta extract-stimulated human mesenchymal stem-cell-derived exosomes. * *p* < 0.001 vs. UV alone.

The collagen, elastin, and hyaluronic acid synthase mRNA levels were measured following treatment with the five additives and UV radiation for 5 or 25 min (Figure 3). UV irradiation decreased collagen mRNA expression levels compared to the non-irradiated group; however, it increased elastin and hyaluronic acid synthase mRNA levels. Pla-Ext increased collagen expression to counteract the response but decreased elastin and hyaluronic acid synthase expression. hMSC-Exo was not resistant to collagen but was resistant to elastin and hyaluronic acid synthase in response to UV radiation. However, co-treatment with Pla-Ext and hMSC-Exo resulted in weakened resistance. Furthermore, the exosomes obtained by stimulating hMSCs with Pla-Ext were resistant to the three parameters.

### 2.3. Damage Resistance in HDFs Subjected to Glycation Stress

**Figure 4:**
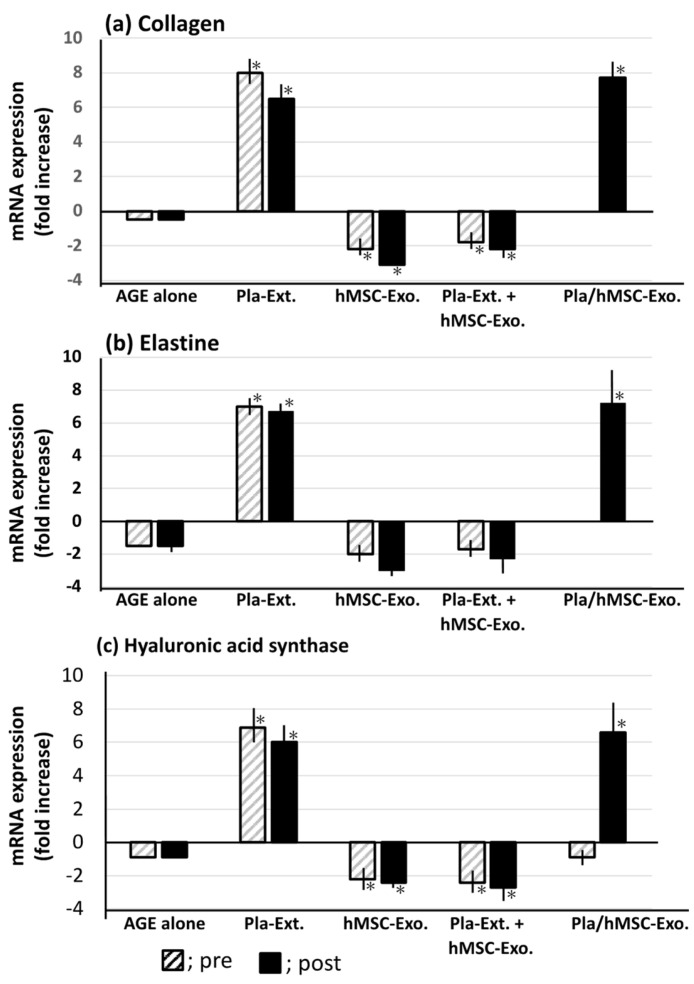
Effects of Pla-Ext on (**a**) collagen, (**b**) elastin, and (**c**) hyaluronic acid synthase mRNA expression levels in human dermal fibroblasts treated with advanced glycation end-products (AGE). Pla-Ext, placental extract; hMSC-Exo, human mesenchymal stem-cell-derived exosomes; Pla extract-stimulated hMSC-Exo; Placenta extract-stimulated human mesenchymal stem-cell-derived exosomes. * *p* < 0.001 vs. AGE alone.

The HDFs tended to display reduced collagen, elastin, and hyaluronic acid synthase mRNA expression levels following treatment with AGEs. This effect was reversed by Pla-Ext treatment, which elevated the expression levels of all three proteins. hMSC-Exo stimulated by Pla-Ext also showed a similar effect. hMSC-Exo showed a tendency to further reduce the mRNA levels that were decreased by AGEs. In addition, when combined with Pla-Ext, they seemed to inhibit the action of Pla-Ext (Figure 4).

### 2.4. Damage Resistance in HDFs Subjected to Oxidative Stress

**Figure 5:**
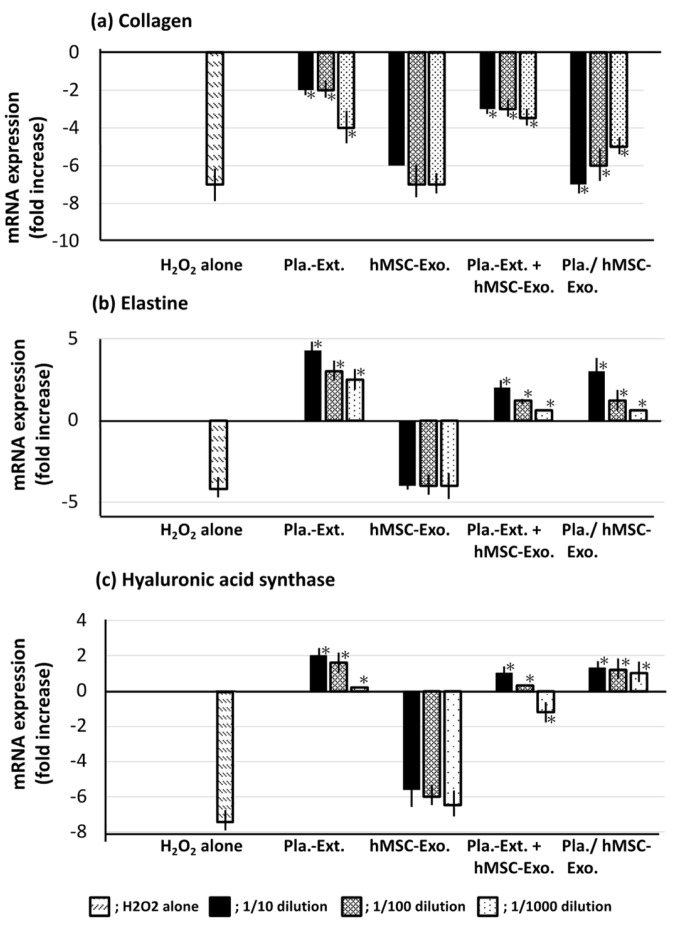
Effects of Pla-Ext on (**a**) collagen, (**b**) elastin, and (**c**) hyaluronic acid synthase in human dermal fibroblasts treated with H_2_O_2_. Pla-Ext, placental extract; hMSC-Exo, human mesenchymal stem-cell-derived exosomes; Pla extract-stimulated hMSC-Exo; Placenta extract-stimulated human mesenchymal stem-cell-derived exosomes. * *p* < 0.001 vs. H_2_O_2_ alone.

H_2_O_2_ stress significantly reduced collagen, elastin, and hyaluronic acid synthase mRNA levels in HDFs, compared with untreated control levels. Pla-Ext mitigated collagen damage and compensated for the reduction in elastin and hyaluronic acid synthase, surpassing untreated control levels. hMSC-Exo alone did not confer resistance. However, Pla-Ext-stimulated hMSC-Exo conferred resistance. When Pla-Ext and hMSC-Exo were used together, the effect of Pla-Ext was almost maintained, and the effect of the combination with hMSC-Exo was almost nonexistent (Figure 5).

### 2.5. Inhibitory Efficacy against Intracellular ROS Accumulation in HDFs Subjected to Oxidative Stress

**Figure 6:**
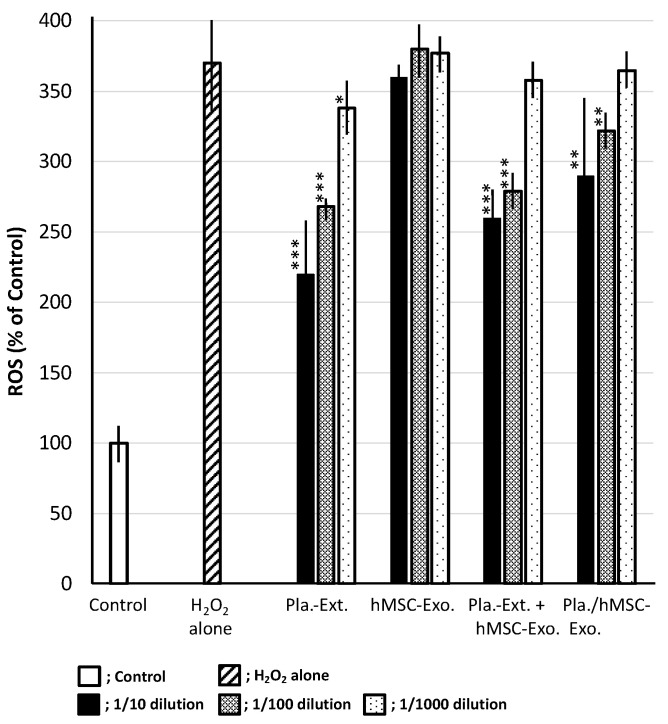
Effects of Pla-Ext on the generation of reactive oxygen species (ROS) in human dermal fibroblasts treated with H_2_O_2_. Pla-Ext, placental extract; hMSC-Exo, human mesenchymal stem-cell-derived exosomes; Pla extract-stimulated hMSC-Exo; placenta extract-stimulated human mesenchymal stem-cell-derived exosomes. * *p* < 0.05, ** *p* < 0.01, *** *p* < 0.001 vs. H_2_O_2_ alone.

H_2_O_2_ stress results in ROS accumulation in HDFs, with an increase of up to 370% when the control is set at 100%. Pla-Ext reduced ROS in a concentration-dependent manner. hMSC-Exo alone did not confer resistance. The effect of Pla-Ext seemed to become apparent upon combination with exosomes. Although hMSC-Exo alone had no inhibitory effect, a suppressive effect on ROS production was observed following treatment with Pla-Ext-stimulated hMSC-Exo (Figure 6).

## 3. Discussion

The initial efficacy of placental extracts (Pla-Ext) and human mesenchymal stem-cell-derived exosomes (hMSC-Exo) against aging-induced stress in human dermal fibroblasts (HDFs) was examined. The effects of Pla-Ext alone, hMSC-Exo alone, the combination of Pla-Ext and hMSC-Exo, and hMSC-Exo recovered from cultures in which Pla-Ext was added to hMSCs (Pla/MSC-Exo) were examined using collagen, elastin, and hyaluronan synthase mRNA levels for each effect. Cells were exposed to photoaging (UV irradiation), glycation (stimulated with advanced glycation end-products; AGE), and oxidation (stimulated with H_2_O_2_) as aging-inducing stressors.

Hydroalcoholic human placental extracts reportedly have growth-promoting effects on skin cells. However, cell proliferation has not been directly observed [13,20]. In the present study, Pla-Ext did not significantly affect intracellular parameters in normal skin fibroblasts, but a pro-proliferative effect was observed (Figure 1 and Figure 2). This difference may be due to differences in animal species or extraction methods.

Placenta itself is a tissue that contains many stem cells. Exosomes are also secreted, but the extract also contains many other cellular proteins and extracellular matrix components, such as collagen, elastine, and hyaluronic acid. There is certainly a wide variety of stimuli that are not present in the single stimulation of stem-cell-derived exosomes used in this study, making it extremely difficult to identify the factors at play.

Pla-Ext conferred HDFs with resistance to UV irradiation and glycation and H_2_O_2_ stimulation. In the present study, we examined three parameters—collagen, elastin, and hyaluronic acid synthase. The results demonstrated that stress-induced damage was repaired beyond the original condition, and the treatment conferred resistance to processes related to cellular senescence. Although a combined effect with hMSC-Exo was not established, we observed that preconditioned stem-cell-derived exosomes, stimulated by Pla-Ext, conferred resistance to senescence-related stress (Figure 3, Figure 4 and Figure 5). hMSC-Exo did not inhibit H_2_O_2_-induced ROS accumulation; however, an inhibitory effect was observed with hMSC-Exo derived from preconditioning with Pla-Ext alone and Pla-Ext (Figure 6).

hMSC-Exo alone tended to improve damage caused by UV irradiation (Figure 3). However, it did not show resistance to AGE, H_2_O_2_ stress, or accumulation of ROS (Figure 4, Figure 5 and Figure 6). On the other hand, exosomes secreted from cultured hMSCs pretreated with Pla-Ext. (Pla./hMSC-Exo.) showed resistance to UV, AGE, and H_2_O_2_ stress (Figure 3, Figure 4 and Figure 5) and were also effective against accumulation of ROS (Figure 6).

This suggests that the mechanisms of aging, such as photoaging and oxidative stress caused by AGEs or H_2_O_2_, are different, and that Pla-Ext treatment may have influenced changes in the surface molecules or content (such as mRNA) of hMSC-secreted exosomes. Several studies have demonstrated that stem cell preconditioning improves the capacity of MSCs, as well as exosome secretion and function, indicating the enhanced biological effects of pretreated MSC exosomes and their improved therapeutic effects against various diseases [21,22,23,24,25,26]. Although Pla-Ext had no specific effect on cultured skin fibroblasts in vivo, Pla-Ext is thought to have some effect when applied to the non-stressed skin of living organisms [3].

This study suggests the following possibilities when Pla-Ext is present in epidermal tissue after application.

Although Pla-Ext does not have any particular effect when it simply reaches fibroblasts, it has been suggested that it may reduce damage caused by external stress, such as ultraviolet radiation or internal glycation and oxidation.

Furthermore, if Pla-Ext reaches skin stem cells, it may provide some kind of stimulus that causes them to secrete exosomes with different characters than the exosomes normally secreted by stem cells.

It will be necessary to investigate in detail in the future what changes occur in stem-cell-derived exosomes pretreated with Pla-Ext. In addition, we plan to verify the effects of Pla-Ext on established senescent cells and the effects of pretreated exosomes.

In the future, it may be possible to infer the anti-skin aging mechanism at the molecular level by analyzing exosome surface molecules and even their cargo (microRNA, etc.).

An increasing number of studies have demonstrated that exosomes alone or in combination with Pla-Ext and preconditioned stem-cell-derived exosomes can secrete a range of trophic factors, including cytokines, growth factors, and chemokines, from MSCs. Therefore, MSCs are considered a paracrine tool [27,28]. Future studies should examine the effects of stem-cell-derived secretome stimulation.

## 4. Materials and Methods

### 4.1. Porcine Placental Extracts (Pla-Ext)

Pla-Exts were fed through a GMP-grading cosmetic manufacturer (Sapporo, Japan). At the manufacturing plant, raw placentas were supplied by a pig farm and stored frozen for several months before entering the manufacturing process, after confirming the absence of infectious disease at the pig farm. Certificates ensuring the safety of the extracts, including sterility tests and the absence of residual estrogens, were issued. The extracts were approved as cosmetic preparations.

### 4.2. Cell Cultures

HDFs and hMSCs were purchased from PromoCell (Heidelberg, Germany) and subcultured in a 5% CO_2_ incubator at 37 °C in a designated, dedicated medium for experimental use.

### 4.3. Isolation of Cultured Cell-Derived Exosomes

hMSC-derived exosomes or Pla/MSC-Exo from hMSC and Pla-Ext-treated hMSC cultures were recovered using a miRCURY Exosome Isolation Kit (Product#: 300102; Exiqon, Hovedstaden, Denmark).

### 4.4. Photoaging (UV) Stimulation

Various cell types were cultured in a 24-well culture plate. Culturing was continued by quickly returning the plate to a CO_2_ incubator at 37 °C after each well was irradiated for 5 or 25 min at a constant distance from the UV lamp. The UV radiation doses were set at 2 J/cm^2^ for 5 min and 10 J/cm^2^ for 25 min using a UV-intensity meter.

### 4.5. AGE Generation

AGE treatment of bovine serum albumin (BSA, Fraction V; Sigma-Aldrich, St. Louis, MO, USA) was performed according to the methods described by Maeda et al. [29]. Briefly, BSA (25 mg/mL) was reacted with 0.2 M phosphate buffer (pH 7.4) for 7 days. Subsequently, the glyceraldehyde that did not bind to BSA was removed using PD-10 gel-filtration columns equilibrated with phosphate-buffered saline. The glycated BSA in the obtained samples was quantified using a Glyceraldehyde-derived AGE-ELISA KIT and subjected to experimentation.

### 4.6. Oxidative Stress Reagent

H_2_O_2_ (FUJIFILM Wako Pure Chemical Corporation, Osaka, Japan) was added to the culture solution by diluting the stock solution with purified water to a final concentration of 0.2 mM [30].

### 4.7. Intracellular Total RNA Extraction

Intracellular total RNA was extracted using a Trizol reagent (Ambion, Austin, TX, USA) according to the manufacturer’s instructions.

### 4.8. RT-qPCR

Collagen [31], elastin [32], and hyaluronic acid synthase [31] mRNA expression levels were determined using a one-step RT-qPCR method with the respective specific primers (Table 1). Specifically, one-step PCR was performed in the same tubing using a Luna Universal One-Step qRT-PCR Kit (New England Biolabs, Ipswich, MA, USA) and Thermal Cycler Dice Real Time System II (Takara Bio, Shiga, Japan). The reaction was performed according to the reagent kit manufacturer’s instructions. Glyceraldehyde-3-phosphate dehydrogenase [31], a constant-expression gene, was used to calculate the delta Ct [33] with internal standards. Differences from the experimental controls were determined using the delta–delta Ct method and expressed as fold changes in mRNA expression.

### 4.9. Determination of Intracellular Reactive Oxygen Species

Intracellular ROS production was determined by a fluorometric assay using dichlorofluorescein-diacetate (DCF-DA; Invitrogen, Carlsbad, CA, USA). After labeling the cells in the experimental groups with 10 mM DCF-DA at 37 °C for 20 min, intracellular ROS was assessed by measuring the excitation wavelength fluorescence intensity (525 nm) using an Agilent Microplate Reader (Agilent Technologies, Santa Clara, CA, USA).

### 4.10. Experimental Protocol

#### 4.10.1. Effects on Normal HDFs

HDFs were treated with purified water (DW), Pla-Ext alone, hMSC-Exo alone, exosomes (Pla/MSC-Exo) secreted from hMSCs stimulated by P-Ext + hMSC-Exo, and Pal-Ext, which were serially diluted (1/100, 1/1000, 1/10,000 dilutions) to extract intracellular total RNA. Collagen, elastin, and hyaluronic acid synthase mRNA expression levels at 1, 4, and 8 h were determined. In addition, we also determined the number of viable cells after 24, 36, and 48 h following the respective stimuli in the five groups.

#### 4.10.2. Resistance to Damage in HDFs Stimulated by Photoaging

HDFs were subjected to the five treatments for 2 h. Subsequently, the treated HDFs were irradiated with UV for 5 or 25 min and further cultured for 2 h. Thereafter, total HDF RNA was extracted, and collagen, elastin, and hyaluronic acid synthase mRNA levels were determined using RT-qPCR.

#### 4.10.3. AGE Receptor mRNA Expression in HDFs Subjected to Glycation Stress

HDFs were subjected to glycation stress by AGE treatment (100 mg/mL) for 2 h. After subjection to the five treatments for 4 h before or after stress, total RNA in HDFs was extracted, and collagen, elastin, and hyaluronic acid synthase mRNA levels were determined using the RT-qPCR method.

#### 4.10.4. Damage Resistance in HDFs Subjected to Oxidative Stress

HDFs were subjected to H_2_O_2_ oxidative stress for 2 h. After subjecting the HDFs to the five treatments for 4 h before or after stress, total HDF RNA was extracted, and collagen, elastin, and hyaluronic acid synthase mRNA levels were determined using the RT-qPCR method. The efficiency of H_2_O_2_ treatment in inhibiting intracellular ROS accumulation was also determined.

### 4.11. Statistical Analysis

All experiments were performed in triplicate, and the data are presented as mean ± SD. Student’s *t*-test was performed, and statistical significance was considered at *p* < 0.05. According to RT-qPCR, differences were considered significant when they differed from delta–delta Ct by more than 2 (≥4-fold difference at mRNA levels).

## 5. Conclusions

In this study, we evaluated the early effects of Pla-Ext and stem-cell-derived exosomes on UV irradiation, glycation, and oxidative stress, which are known as aging stresses in skin cells. Pla-Ext has been shown to have the potential to increase resistance to early aging stress by acting directly on fibroblasts. Furthermore, this resistance was also achieved by exosomes secreted from cultured hMSCs pretreated with Pla-Ext (Pla/MSC-Exo). It is a new discovery that an effect that cannot be obtained by exosomes constantly secreted from hMSCs was obtained by stimulation with Pal-Ext. It was suggested that Pla-Ext penetrates the epidermis and acts directly on fibroblasts. In addition, it was proposed that some kind of stimulation of dermal stem cells may have some effect on the secreted exosomes, enabling resistance to early aging stress. This led to the analysis of a new mechanism, where Pla-Ext directly reaches and acts on dermal stem cells.

## Figures and Tables

**Table 1:** Primers for RT-qPCR.

Gene	Primer	Ref.
Collagen 1 A2	Forward: CTGGACCTCCAGGTGTAAGC	[29]
	Reverse: TGGCTGAGTCTCAAGTCACG	
Elastin	Forward: GGCCATTCCTGGTGGAGTTCC	[30]
	Reverse: AACTGGCTTAAGAGGTTTGCCTCCA	
Hyaluronic acid synthase	Forward: CACGTAACGCAATTGGTCTTGTCC	[29]
	Reverse: CCAGTGCTCTGAAGGCTGTGTAC	
GAPDH	Forward: GACATGCCGCCTGGAGAAAC	[29]
	Reverse: AGCCCAGGATGCCCTTTAGT	

## Data Availability

The original contributions presented in the study are included in the article, further inquiries can be directed to the corresponding author.

## References

[1] Shin S.H., Lee Y.H., Rho N.K., Park K.Y. (2023). Skin aging from mechanisms to interventions: Focusing on dermal aging. Front. Physiol..

[2] Wang Y., Branicky R., Noë A., Hekimi S. (2018). Superoxide dismutases: Dual roles in controlling ROS damage and regulating ROS signaling. J. Cell Biol..

[3] Lv J., Yang S., Lv M., Lv J., Sui Y., Guo S. (2022). Protective roles of mesenchymal stem cells on skin photoaging: A narrative review. Tissue Cell.

[4] Chen B., Sun Y., Zhang J., Zhu Q., Yang Y., Niu X., Deng Z., Li Q., Wang Y. (2019). Human embryonic stem cell-derived exosomes promote pressure ulcer healing in aged mice by rejuvenating senescent endothelial cells. Stem Cell Res. Ther..

[5] Yang A., Shuyan L., Xiaojie T., Shiou Z., Fangfei N., Yonghuan Z., Luosha G., Chunlei Z., Baicheng W., Wei W. (2021). Exosomes from adipose-derived stem cells and application to skin wound healing. Cell. Prolif..

[6] Zeng W., Guo L. (2021). Research Advances in the Application of Adipose-Derived Stem Cells Derived Exosomes in Cutaneous Wound Healing. Ann. Dermatol..

[7] Hong J.W., Lee W.J., Hahn S.B., Kim B.J., Lew D.H. (2010). The effect of human placenta extract in a wound healing model. Ann. Plast. Surg..

[8] Nath S., Bhattacharyya D. (2007). Cell adhesion by aqueous extract of human placenta used as wound healer. Indian J. Exp. Biol..

[9] Pan S.Y., Chan M., Wong M., Klokol D., Chernykh V. (2017). Placental therapy: An insight to their biological and therapeutic properties. J. Med. Ther..

[10] Kong M.H., Lee E.J., Lee S.Y., Cho S.J., Hong Y.S., Park S.B. (2008). Effect of human placental extract on menopausal symptoms, fatigue, and risk factors for cardiovascular disease in middle-aged Korean women. Menopause.

[11] Togashi S., Takahashi N., Iwama M., Watanabe S., Tamagawa K., Fukui T. (2002). Antioxidative collagen-derived peptides in human-placenta extract. Placenta.

[12] Yoshikawa C. (2013). Effect of porcine placental extract on collagen production in human skin fibroblasts in vitro. Gynecol. Obstet..

[13] Tonello G., Daglio M., Zaccarelli N., Sottofattori E., Mazzei M., Balbi A. (1996). Characterization and quantitation of the active polynucleotide fraction (PDRN) from human placenta, a tissue repair stimulating agent. J. Pharm. Biomed. Anal..

[14] Takuma K., Mizoguchi H., Funatsu Y., Kitahara Y., Ibi D., Kamei H., Matsuda T., Koike K., Inoue M., Nagai T. (2012). Placental extract improves hippocampal neuronal loss and fear memory impairment resulting from chronic restraint stress in ovariectomized mice. J. Pharmacol. Sci..

[15] Lee K.H., Park H.J., Seo H.G., Kim J.H., Lim G.S., Lee W.Y., Kim N.H., Kim J.H., Lee J.H., Jung H.S. (2013). Immune modulation effect of porcine placenta extracts in weaned the pig. J. Anim. Sci..

[16] Choi H.Y., Kim S.W., Kim B., Lee H.N., Kim S.J., Song M., Kim S., Kim J., Kim Y.B., Kim J.H. (2014). Alpha-fetoprotein, identified as a novel marker for the antioxidant effect of placental extract, exhibits synergistic antioxidant activity in the presence of estradiol. PLoS ONE.

[17] Matsuoka T., Takanashi K., Dan K., Yamamoto K., Tomobe K., Shinozuka T. (2021). Effects of mesenchymal stem cell-derived exosomes on oxidative stress responses in skin cells. Mol. Biol. Rep..

[18] Eckes B., Mauch C., Hüppe G., Krieg T. (1996). Differential regulation of transcription and transcript stability of pro-alpha 1 (I). Biochem. J..

[19] Hwang K.A., Yi B.R., Choi K.C. (2011). Molecular mechanisms and in vivo mouse models of skin aging associated with dermal matrix alterations. Lab. Anim. Res..

[20] Pal P., Roy R., Datta P.K., Dutta A.K., Biswas B., Bhadra R. (1995). Hydroalcoholic human placental extract: Skin pigmenting activity and gross chemical composition. Int. J. Dermatol..

[21] Phan J., Kumar P., Hao D., Gao K., Farmer D., Wang A. (2018). Engineering mesenchymal stem cells to improve their exosome efficacy and yield for cell-free therapy. J. Extracell. Vesicles.

[22] Cha J.M., Shin E.K., Sung J.H., Moon G.J., Kim E.H., Cho Y.H., Park H.D., Bae H., Kim J., Bang O.Y. (2018). Efficient scalable production of therapeutic microvesicles derived from human mesenchymal stem cells. Sci. Rep..

[23] Ferreira J.R., Teixeira G.Q., Santos S.G., Barbosa M.A., Almeida-Porada G.A., Gonçalves R.M. (2018). Mesenchymal stromal cell secretome: Influencing therapeutic potential by cellular pre-conditioning. Front. Immunol..

[24] Pendse S., Kale V., Vaidya A. (2021). Extracellular vesicles isolated from mesenchymal stromal cells primed with hypoxia: Novel strategy in regenerative medicine. Curr. Stem Cell Res..

[25] Yu B., Kim H.W., Gong M., Wang J., Millard R.W., Wang Y., Ashraf M., Xu M. (2015). Exosomes secreted from GATA-4 overexpressing mesenchymal stem cells serve as a reservoir of anti-apoptotic microRNAs for cardioprotection. Int. J. Cardiol..

[26] Liang L., Zheng D., Lu C., Xi Q., Bao H., Li W., Gu Y., Mao Y., Xu B., Gu X. (2021). Exosomes derived from miR-301a-3p-overexpressing adipose-derived mesenchymal stem cells reverse hypoxia-induced erectile dysfunction in rat models. Stem Cell Res. Ther..

[27] Mazini L., Rochette L., Admou B., Amal S., Malka G. (2020). Hopes and limits of adipose-derived stem cells (ADSCs) and mesenchymal stem cells (MSCs) in wound healing. Int. J. Mol. Sci..

[28] Gnecchi M., He H., Noiseux N., Liang O.D., Zhang L., Morello F., Mu H., Melo L.G., Pratt R.E., Ingwall J.S. (2006). Evidence supporting paracrine hypothesis for Akt-modified mesenchymal stem cell-mediated cardiac protection and functional improvement. FASEB J..

[29] Maeda S., Matsui T., Ojima A., Takeuchi M., Yamagishi S.I. (2014). Sulforaphane inhibits advanced glycation end product-induced pericyte damage by reducing expression of receptor for advanced glycation end products. Nutr. Res..

[30] Kartal B., Akçay A., Palabiyik B. (2018). Oxidative stress upregulates the transcription of genes involved in thiamine metabolism. Turk. J. Biol..

[31] Liu W., Ma C., Li H.Y., Chen L., Yuan S.S., Li K.J. (2020). MicroRNA-146a downregulates the production of hyaluronic acid and collagen I in Graves’ ophthalmopathy orbital fibroblasts. Exp. Ther. Med..

[32] Deslee G., Woods J.C., Moore C.M., Liu L., Conradi S.H., Milne M., Gierada D.S., Pierce J., Patterson A., Lewit R.A. (2009). Elastin expression in very severe human COPD. Eur. Respir. J..

[33] Livak K.J., Schmittgen T.D. (2001). Analysis of relative gene expression data using real-time quantitative PCR and the 2^−ΔΔCt^ method. Methods.

